# Recycling of Citric Acid Waste for Potential Use as Animal Feed through Fermentation with Lactic Acid Bacteria and a Mixture of Fibrolytic Enzymes

**DOI:** 10.3390/ani12213049

**Published:** 2022-11-06

**Authors:** Sirisak Tanpong, Sawitree Wongtangtintharn, Anusorn Cherdthong, Rittikeard Prachumchai, Bundit Tengjaroenkul, Pin Chanjula, Chanon Suntara, Chalong Wachirapakorn

**Affiliations:** 1 Department of Animal Science, Faculty of Agriculture, Khon Kaen University, Khon Kaen 40002, Thailand; 2 Department of Veterinary Public Health, Faculty of Veterinary Medicine, Khon Kaen University, Khon Kaen 40002, Thailand; 3 Animal Production Innovation and Management Division, Faculty of Natural Resources, Hat Yai Campus, Prince of Songkla University, Songkhla 90110, Thailand

**Keywords:** waste, improvement, animal feed, environmental impact, recycle

## Abstract

**Simple Summary:**

The by-products have the advantage of being converted into inexpensive animal feed additives, which lowers the cost of animal feed. We hypothesized that citric acid by-product (CAP) might be used for animal feed if sufficient quality improvement occurred, which would lessen environmental impact. We discovered that employing inoculants with fibrolytic enzymes and lactic acid bacteria (*Lactobacillus casei* TH14) improves the quality of CAP. By reducing the percentage of crude fiber, neutral detergent fiber, and acid detergent fiber at 28 days, this combination is very effective for improving CAP characteristics. Combining *L. casei* TH14with fibrolytic enzymes is the most efficient strategy to lower crude fiber and pH and improve carbohydrate breakdown.

**Abstract:**

Once improperly managed, the citric acid production industry generates waste, which contributes to pollution and other environmental issues. We proposed that, with sufficient quality improvement, citric acid by-product (CAP) might be used for animal feed, thereby reducing the environmental impact. The aim of the present study was to ferment citric acid by-product (CAP) by inoculation with lactic acid bacteria (LAB) and a fibrolytic enzyme mixture for quality improvement and crude fiber reduction in the waste products. LAB inoculants were *L. casei* TH14, and the additive enzyme used was a fibrolytic enzyme mixture (glucanase, pectinase, and carboxymethylcellulase) of a small-scale fermentation method. The seven treatments employed in this study were as follows: (1) control (untreated), (2) CAP-inoculated *L. casei* TH14 at 0.01% DM, (3) CAP-inoculated *L. casei* TH14 at 0.05% DM, (4) CAP-inoculated enzymes at 0.01% DM, (5) CAP-inoculated enzymes at 0.05% DM, (6) CAP-inoculated *L. casei* TH14 at 0.01% DM with enzymes at 0.01% DM, and (7) CAP-inoculated *L. casei* TH14 at 0.05% DM with enzymes at 0.05% DM. The samples were taken on days 1, 7, 14, 21, and 28 of ensiling, both before and after. Four replications were used. The results of the chemical composition of the CAP before and after ensilage inoculated with *L. casei* TH14 did not show any differences in crude protein, ether extract, ash, or gross energy, but the enzymes significantly (*p* < 0.05) decreased crude fiber and increased nitrogen-free extract. The combination was especially effective at improving the characteristics of CAP, with a reduction in crude fiber from 21.98% to 22.69%, of neutral detergent fiber (NDF) from 16.01% to 17.54%, and of acid detergent fiber (ADF) from 13.75% to 16.19%. Furthermore, the combination of *L. casei* TH14 and the enzyme increased crude protein from 1.75% to 2.24% at 28 days of ensiling. Therefore, CAP-inoculated *L. casei* TH14 did not change in chemical composition, while crude fiber, NDF, and ADF decreased when CAP was inoculated with enzyme. The combination of *L. casei* TH14 and the enzyme is more effective at improving chemical composition and reducing crude fiber and enhancing carbohydrate breakdown in the CAP. Finally, by enhancing the CAP’s quality, it may be possible to use it in animal feed and minimize its impact on the environment.

## 1. Introduction

Citric acid is the most important source of organic acids and the second-largest fermentation product in the world, producing more than 1.7 million tons per year. Due to the wide applications of citric acid, it is estimated that the global market demand for citric acid, as measured by the production of citric acid, increases at a rate of 5% every year [1]. It was discovered that between 50 and 60% of the substrate was wasted during the manufacture of citric acid worldwide [2]. The citric acid production industry produces waste products and causes pollution and other environmental problems when not managed properly [1]. Animal feed is becoming an increasingly important component of animal production demand [3]. The by-products include cellulose, hemicellulose, sugars, starches, and protein; all of these ingredients should be used to make animal feed. It is challenging to be using by-products of the citric acid manufacturing industry as animal feed. The benefits of by-products include the ability to produce low-cost products while also reducing pollution and environmental issues [4].

The data show that the chemical composition of the citric acid by-product (CAP) consists of 7–9% moisture content, 11–19% ash, 7–8% crude protein (CP), 0.8–1.2% ether extract (EE) content, 18–23% crude fiber (CF), and 13.06–14.15 MJ/kg DM of gross energy (GE) [5,6]. In their research on the CAP in ruminants, Suntara and Uriyapongson [7] looked at CAP fermentation with exogenous fibrolytic enzymes and included diets for culled beef cattle at a rate of 30%. They found no significant effects on growth performance, but the feed cost was lower compared to the control group. Similar findings were made by Chanvech and Wachirapakorn [8], who found that beef cattle’s feed intake and digestibility may be increased by supplementing CAP with a fibrolytic enzyme at a level of 10% of the diet. Many studies have focused on ruminant animals, and very little data have been elucidated for non-ruminant species. In any case, since non-ruminants are unable to consume high-fiber feed, lowering fiber content and increasing nutritional value should be examined. 

In theory, dietary fiber has effects on productivity in non-ruminant animals. High-fiber diets generate physical distension of the walls of the gastrointestinal tract (GIT), increasing GIT capacity related to gut fill and having negative effects on nutrient digestibility [9]. In recent years, microbial inoculants have been used in the fermentation of non-starch polysaccharides and dietary fiber. Lactic acid bacteria (LAB), *Streptococcus*, *Bifidobacterium*, and *Bacillus* were used in an effort to microbial ferment the dietary fiber component [10]. Lactic acid bacteria are commonly used for the preservation of food and animal feed. They also create lactic acid, organic acids, hydrogen peroxide, and bacteriocins [11]. Finally, the generation of lactic acid during lactic acid fermentation significantly lowers the pH, which appears to inhibit the growth of Gram-negative pathogenic bacteria [12,13]. When forecasting the effectiveness of fermentation and deciding whether to apply bacterial inoculants to silage, the quantity and features of LAB have grown to be a crucial consideration. In order to improve fermentation quality, many LAB-containing biological additives have been developed and are currently available [11]. The fibrolytic enzyme was commonly used as a supplement to silage for partially degrading fiber into digestible, water-soluble carbohydrates that were then used by LAB because some microbial organisms cannot use fiber as an energy source. Enzyme cellulase improves fiber degradation, increasing water soluble carbohydrates (WSC) as a substrate for LAB to produce lactic acid [11]. 

We postulated that if there were proper quality improvements, which would reduce environmental impact, CAP might be used for animal feed. Therefore, the aim of this study is to improve the quality of CAP and reduce crude fiber by fermenting CAP from industry with *L. casei* TH14 and a fibrolytic enzyme mixture. 

## 2. Materials and Methods

### 2.1. Sampling

The study was conducted at the Department of Animal Science, Faculty of Agriculture and Science, Khon Kaen University, Thailand. Random survey sampling was used to collect the waste products from citric acid producers in the eastern region of Thailand. The randomly collected samples were then handled carefully to maintain their original integrity and the nutrient compositions of the waste products for investigation.

### 2.2. Sample Preparation and Experiments

In order to study citric acid fermentation to evaluate the effect of moisture adjustment on fermentation quality, 50% of the waste products were ensiled using small-scale plastic bag fermentation [11]. One hundred grams were packed into plastic film bags that were then vacuum-sealed. The *Lactobacillus casei*-using lactic acid bacteria strains that Pholsen et al. [14] provided were identified and described. They were obtained from tropical forages and associated silages. The selected strains were isolated from a silage prepared with sweet corn (*Zea mays* L.) stover, sugar cane (*Saccharum officinarum* L.) top, and rice straw (*Oryza sativa* L.). These strains were used as additives at 1.0 × 10^5^ colony-forming units (cfu/g). The fibrolytic enzyme mixture used (acremonium cellulase [AC], Meiji Seika Pharma, Tokyo, Japan) was produced by *Acremonium cellulolyticus*. The main components were glucanase and pectinase, and the carboxymethyl cellulase activity was 7350 U/g. The seven treatments employed in this study were as follows: (1) control (untreated), (2) CAP-inoculated *L. casei* TH14 at 0.01% DM, (3) CAP-inoculated *L. casei* TH14 at 0.05% DM, (4) CAP-inoculated enzymes at 0.01% DM, (5) CAP-inoculated enzymes at 0.05% DM, (6) CAP-inoculated *L. casei* TH14 at 0.01% DM with enzymes at 0.01% DM, and (7) CAP-inoculated *L. casei* TH14 at 0.05% DM with enzymes at 0.05% DM, respectively. The silages were kept at room temperature (25 °C). The samples from before and after ensiling with four replications at 1, 7, 14, 21, and 28 days of ensiling were used for the chemical composition and pH value analyses. The silage length period was chosen based on Chanvech and Wachirapakorn [8]’s recommendation that 28 days of fermentation would successfully improve CAP quality. 

### 2.3. Nutrient Compositions 

Silage substrates were dried at 60 °C and ground to pass through a 1-mm filter (Cyclotech Mill, Tecator, Hoganas, Sweden). The chemical composition of silage ground samples was determined. The Association of Official Analytical Chemists (AOAC) method was used to determine the DM (100 °C for 24 h), CP (distillation Kjeldahl), CF, ash content (550 °C for 6 h), and EE of the samples. Based on proximate nutritional values, the nitrogen-free extract (NFE) content was calculated. Bomb calorimeters were used to measure GE with adiabatic calorimeters (AC 500, Leco, St. Joseph, MI, USA). The pH measurement was evaluated according to Cai [11]: the 10-gram sample was centrifuged with 90 mL of distilled water and measured with a pH meter (pHep Hi 98128, Hanna Instrument, Curepipe, Mauritius). The pH meter was calibrated with buffers at a pH of 4.0, 7.0, and 10. Neutral detergent fiber (NDF) and acid detergent fiber (ADF) were analyzed according to Van Soest et al. [15] by filter bag technique. The acid detergent lignin (ADL) was analyzed according to Faichney and White [16].

### 2.4. Statistical Analysis

Chemical compositions and pH were analyzed by one-way ANOVA using the procedure of SAS (SAS Institute Inc., Cary, NC, USA). A completely randomized design (CRD) and a CRD with 7 × 5 (additive types [A] × day fermentation types [B]) in factorial treatments revealed significant differences, as determined by Duncan’s new multiple-range test. Statistical significance was set at *p* < 0.05.

## 3. Result and Discussion

### 3.1. Nutrient Composition of Citric Acid By-Product before Ensiling

The nutrient composition of CAP, as determined by proximate analysis, is presented in Table 1. Before ensiling, the chemical composition was 92.70% DM, 7.30%moisture, 13.21% ash, 10.32% insoluble ash, 2.89% soluble ash, 6.11% CP, 2.39% EE, 18.26% CF, 0.90% calcium, 0.08% phosphorus, and 15.01 MJ/kg DM of gross energy. The citric acid content and pH of the CAP were 0.71% and 4.68, respectively.

### 3.2. Nutrient Composition of Citric Acid By-Product after Ensiling with Lactic Acid Bacteria and an Enzyme

The experiments included untreated (control), treated *L. casei* TH14, fibrolytic enzymes, and combined *L. casei* TH14 and fibrolytic enzymes. The results are presented in Table 2, Table 3 and Table 4. The nutritional contents of ash, CP, EE, CF, calcium, phosphorus, gross energy, NDF, ADF, ADL, and pH were not significantly different among the treatments at 1 day of ensiling (*p* > 0.05). The seven days of ensiling the CAP did not yield any significant differences in ash, CP, EE, CF, calcium, phosphorus, or gross energy. However, the fermentation with *L. casei* TH14 and the fibrolytic enzymes in combination resulted in a significantly lower pH compared with controls (*p* < 0.05). There was a significant interaction effect between treatments and ensiling time on CF, NFC, NDF, and ADF (*p* < 0.01). The fermentation at 14, 21, and 28 days of ensiling with enzymes and with a combination of *L. casei* TH14 and the fibrolytic enzymes at the levels of 0.01% and 0.05% decreased CF compared with the control group. The 0.05% fibrolytic enzymes raised NFE by 1.5 to 2.44%. CAP fermented using a combination of *L. casei* TH14 at 0.05% DM and enzyme at 0.05% DM had the lowest NDF and ADF content. Furthermore, ensilage CAP for 28 days can reduce 3.2% NDF and 1.57% ADF when compared to day 1 of fermentation. Although all treatments significantly reduced pH at all time points, the exact reduction varied between treatments at different time points. Furthermore, the pH of the CAP combination of *L. casei* TH14 at 0.05% DM and enzyme at 0.05% DM was substantially lower (*p* < 0.05) than the other treatments (Table 5). The CAP-inoculated with *L. casei* TH14 and fibrolytic enzymes in combination showed a significant (*p* < 0.01) decrease in CF, NDF, and ADF at 14, 21, and 28 days compared with 1 and 7 days. 

Our study investigated whether *L. casei* TH14 isolated from tropical forages and their silages could be used for reducing the CF of the CAP from the citric acid industry. The findings demonstrated that the level of *L. casei* TH14 fermentation inoculation had no impact on the content of nutrients. The CAP from ensiling experienced a reduction in CF and an increase in NFE following the inoculation of *L. casei* TH14 in combination with fibrolytic enzymes. The basic idea of silage is to store forage for increased stability and nutritional value until its use as animal feed. Lactic acid bacteria are commonly used for the preservation of food and animal feed. Lactic acid bacteria are extensively employed in silage because of their potential to speed up lactic acid formation, lower pH, prevent protein breakdown, minimize dry matter (DM) loss, and improve animal performance [11,12,13]. 

Considering combination silages, the beneficial impact of adding unique components into the silo on ensiling should be assured in order to minimize unanticipated spoiling, according to Kaewpila et al. [17], who evaluated the *L. casei* TH14 inoculant for preservation on various feed crops and grass, resulting in improved silage quality. The results showed that adding *L. casei* TH14 inoculant to these plant silages might promote lactic acid production and alter in vitro digestibility [17]. The by-products typically contain a high concentration of carbohydrates. Hence, the presence of the lactic acid fermentation process may be a suitable alternative for the process of converting waste products into animal feed. Yang et al. [18] observed that food waste fermented with *L. salivarius* at levels of 0.1%, 0.2%, 0.5%, and 1.0% had no differences in the nutritional content of CP after 30 days of ensiling compared to the treatments conducted before ensiling and the *L. salivarius* inoculation. This was due to the preservation of food waste (15.9 vs. 16.1% CP). However, compared to the control group, it significantly (*p* < 0.05) enhanced the WSC (water soluble carbohydrate) on the treatments inoculated with *L. casei* TH14. Ni et al. [19] studied the effects of inoculation of LAB (*L. plantarum*) isolated from corn and grass and found that inoculation at 30 days of ensiling did not result in significant differences in the chemical composition of EE relative to controls, but significantly (*p* < 0.05) decreased crude fiber by 10.92–12.59% in wheat silages. Similarly, Ando et al. [20] discovered that introducing *L. plantarum* to silage at a concentration of 0.02% fresh matter of guinea grass that had been kept for 45 days had no adverse effect on the chemical composition of CP and EE. Many researchers have conducted studies of LAB inoculation to improve the fermentation characteristics of wheat [21], alfalfa [22,23], grass–legume forage [24], corn forage [22], and brewer’s grains [25]. However, other studies have reported that microbial fermentation did not affect the NDF and ADF content of the forage and by-product silage [22,24,25].

Since certain LAB cannot use fiber as a source of energy. Thus, the addition of fibrolytic enzyme can partially break down fiber into water-soluble carbohydrates, resulting in the availability of LAB growth substrate [26,27,28]. In comparison to the LAB treatments without the enzyme and the control group, the results of the current investigation showed substantial improvements in the nutritional profile, decreased CF, improved NFE, and the stability of low pH in silage. Extracellular enzymes from microorganisms hydrolyze the complex organic component, which includes protein, lipids, and carbohydrates, into smaller soluble molecules. These substrates can be fermented to produce lactic acid, resulting in a lower pH in silage [27]. The soluble sugars are converted into lactic acid by LAB, and the increase in WSC and NFE indicates that carbohydrates are broken down into WSC and NFE [29]. Our results are corroborated by those of Dehghani et al. [28], who found that the fibrolytic enzyme complex was added to maize stover silages and ensiled for 60 days. In comparison to the control group, the CAP combination of *L. casei* TH14 at 0.05% DM and enzyme at 0.05% DM was the most efficient in lowering pH and decreasing NDF by 3.11% (Table 5). These enzymes can degrade the carbohydrate cell walls of NDF and decrease NDF in silages. This demonstrated that the enzymes were more efficient in metabolizing NDF, which is in accordance with the reports of Higginbotham et al. [30], Zhao et al. [23], Shepherd and Kung [26], and Khota et al. [31], who reported on supplementing the commercial cellulase enzyme (*Acremonium cellulolyticus*) with LAB (*L. plantarum*) fermentation in purple Guinea and Napier grass silages at 30 days of ensiling. The results showed that adding 1% fibrolytic enzymes and combining fibrolytic enzymes and *L. plantarum* considerably enhanced CP (15.57 and 12.21%) and decreased NDF (4.73 and 8.31%), respectively. At the end of 90 days of ensiling, Konca et al. [32] discovered that adding commercial LAB (*L. plantarum* and *Enterococcus faecium*) along with the enzymes cellulase and amylase to sunflower silage had no impact on the chemical composition of CP, EE, NDF, ADF, and hemicellulose but significantly (*p* < 0.05) reduced cellulose by 1.86%. Similarly, Hou et al. [33] used the combination of LAB (*L. plantarum*) and cellulolytic enzymes to improve the fermentation quality of natural grasses. The outcomes showed that LAB and enzyme inoculation had positive effects. The combination of LAB and enzyme is beneficial for decreasing CF and pH and boosting the carbohydrate content of the CAP.

## 4. Conclusions

Waste products from the manufacturing of citric acid have a chemical composition that is suitable for fermentation, with a particularly high crude fiber content of 18.26%. Nutritional values are improved by using inoculants that include fibrolytic enzymes and LAB. This combination is particularly efficient at enhancing CAP qualities by lowering the proportion of crude fiber, NDF, and ADF at 28 days. The most effective way to decrease CF, pH, and carbohydrate breakdown is to combine LAB with fibrolytic enzymes. Finally, improving the nutritional quality of CAP may make it feasible to use it in animal feed, thereby reducing the environmental impact. However, more research is needed to verify the animal responses.

## Figures and Tables

**Table 1:** Nutrient composition of citric acid by-product from cassava.

Chemical Compositions	Citric Acid By-Product
Dry matter, %	92.70
Moisture, % DM	7.30
Ash, % DM	13.21
Soluble ash, % DM	2.89
Insoluble ash, % DM	10.32
Crude protein, % DM	6.11
Ether extract, % DM	2.39
Crude fiber, % DM	18.26
Nitrogen-free extract, % DM	52.73
Calcium, % DM	0.90
Phosphorus, % DM	0.08
Gross energy, Megajoule/kg DM	15.01
pH	4.68

**Table 2:** Nutrient composition and pH of citric acid by-product at 1 and 7 days of fermentation.

Day	Trt ^1^	CP, %	Ash, %	EE, %	CF, %	NFE, %	Ca, %	P, %	GE, MJ/kg	NDF, %	ADF, %	ADL, %	pH
1 d	Con	6.27	13.37	2.33	18.21	52.53	0.82	0.08	14.95	39.94	20.32	7.81	4.59
	LAB1	6.22	13.18	2.33	18.24	52.69	0.83	0.08	15.03	39.31	20.03	7.79	4.60
	LAB5	6.24	13.20	2.39	18.01	52.92	0.81	0.08	14.90	39.94	20.12	7.95	4.61
	E1	6.32	13.32	2.35	18.22	52.49	0.80	0.07	15.07	39.31	19.63	8.46	4.60
	E5	6.29	13.43	2.40	18.23	51.94	0.79	0.07	14.91	39.94	20.19	8.04	4.56
	LAB1 + E1	6.22	13.33	2.34	18.18	52.46	0.85	0.08	15.07	39.31	19.69	8.62	4.61
	LAB5 + E5	6.22	13.45	2.36	18.09	52.56	0.83	0.08	15.08	39.31	20.09	8.40	4.64
	*p*-value	0.93	0.93	0.99	0.79	0.49	0.79	0.79	0.86	0.73	0.41	0.75	0.87
	SEM	0.08	0.19	0.08	0.12	0.30	0.03	0.01	3.01	0.44	0.24	0.45	6.64
7 d	Con	6.21	13.30	2.41	18.33	52.43	0.82	0.08	15.01	39.63	20.51	8.25	4.31 ^a^
	LAB1	6.20	13.25	2.61	17.79	52.81	0.88	0.09	15.05	39.65	20.00	7.87	4.16 ^b^
	LAB5	6.13	13.72	2.41	17.89	52.80	0.86	0.08	14.96	40.12	19.63	7.90	4.07 ^bc^
	E1	6.30	13.24	2.49	17.83	52.80	0.88	0.09	15.05	39.53	20.03	8.45	4.06 ^bc^
	E5	6.19	13.44	2.46	17.69	52.89	0.83	0.08	14.97	39.99	19.48	8.48	4.11 ^b^
	LAB1 + E1	6.23	13.81	2.42	17.81	52.74	0.84	0.07	14.92	39.31	19.69	7.99	4.06 ^bc^
	LAB5 + E5	6.29	13.34	2.36	17.49	53.74	0.89	0.09	14.94	39.41	19.83	8.11	3.97 ^c^
	*p*-value	0.92	0.47	0.84	0.27	0.90	0.34	0.11	0.84	0.53	0.22	0.38	<0.001
	SEM	0.10	0.23	0.12	0.21	0.69	0.02	0.10	1.84	0.32	0.27	0.23	0.04

^a,b,c^ Means in the same column without common letter are different at *p* < 0.05. CP = crude protein, EE = ether extract, CF = crude fiber, NFE = nitrogen-free extract, Ca = calcium, P = phosphorus, GE = gross energy, NDF = neutral detergent fiber, ADF = acid detergent fiber, ADL = acid detergent lignin. ^1^ Con = control, LAB1 = LAB 0.01% DM, LAB5 = LAB 0.05% DM, E1 = enzyme 0.01% DM, E5 = enzyme 0.05% DM, LAB1 + E1 = combination LAB 0.01% DM with enzyme 0.01% DM and LAB5 + E5 = combination LAB 0.05% DM with enzyme 0.05% DM.

**Table 3:** Nutrient composition and pH of citric acid by-product at 14 and 21 days of fermentation.

Day	Trt ^1^	CP, %	Ash, %	EE, %	CF, %	NFE, %	Ca, %	P, %	GE, MJ/kg	NDF, %	ADF, %	ADL, %	pH
14 d	Con	6.28	13.21	2.33	18.32 ^a^	52.11 ^bc^	0.81	0.08	15.03	39.74 ^a^	20.44 ^a^	8.57	4.22 ^a^
	LAB1	6.29	13.97	2.74	17.79 ^a^	51.86 ^c^	0.94	0.09	14.97	39.67 ^a^	19.98 ^a^	7.64	3.99 ^b^
	LAB5	6.27	13.80	2.55	17.89 ^a^	52.15 ^bc^	0.88	0.08	15.24	39.92 ^a^	19.80 ^ab^	7.77	3.85 ^c^
	E1	6.25	13.32	2.41	16.97 ^b^	53.64 ^ab^	0.84	0.08	14.97	38.53 ^b^	18.86 ^bc^	8.45	3.94 ^bc^
	E5	6.31	13.32	2.39	16.82 ^b^	54.30 ^a^	0.85	0.08	14.92	37.66 ^b^	18.64 ^c^	8.66	3.93 ^bc^
	LAB1 + E1	6.42	13.78	2.47	16.04 ^c^	54.44 ^a^	0.90	0.08	14.86	38.03 ^b^	18.79 ^c^	8.05	3.95 ^bc^
	LAB5 + E5	6.40	12.85	2.50	15.82 ^c^	55.10 ^a^	0.94	0.08	14.95	37.85 ^b^	18.76 ^c^	8.11	3.81 ^c^
	*p*-value	0.72	0.19	0.20	<0.001	0.001	0.55	0.81	0.28	<0.001	0.001	0.06	<0.001
	SEM	0.09	0.30	0.10	0.22	0.50	0.06	0.07	2.44	0.31	0.31	0.24	0.04
21 d	Con	6.21	13.38	2.30	18.31 ^a^	52.09 ^b^	0.80	0.08	14.99	39.80 ^a^	20.44 ^a^	8.67	4.26 ^a^
	LAB1	6.19	13.37	2.40	18.32 ^a^	50.01 ^c^	0.87	0.08	14.70	39.97 ^a^	19.98 ^a^	7.97	3.89 ^b^
	LAB5	6.16	13.40	2.38	18.15 ^a^	51.80 ^bc^	0.88	0.08	14.77	39.63 ^a^	19.80 ^a^	7.90	3.87 ^b^
	E1	6.35	13.50	2.44	16.22 ^b^	53.76 ^ab^	0.84	0.08	14.92	36.83 ^b^	17.86 ^b^	8.21	3.89 ^b^
	E5	6.50	13.11	2.45	15.64 ^bc^	53.63 ^ab^	0.87	0.07	14.83	34.39 ^c^	16.89 ^c^	8.89	3.83 ^bc^
	LAB1 + E1	6.50	13.15	2.34	15.68 ^bc^	54.21 ^a^	0.89	0.08	14.97	36.04 ^b^	17.16 ^bc^	7.96	3.78 ^bc^
	LAB5 + E5	6.44	13.43	2.51	15.03 ^c^	55.37 ^a^	0.87	0.09	14.91	33.99 ^c^	16.86 ^c^	8.00	3.71 ^c^
	*p*-value	0.33	0.38	0.96	<0.001	<0.001	0.69	0.27	0.22	<0.001	<0.001	0.11	<0.001
	SEM	0.13	0.15	0.15	0.34	0.64	0.04	0.01	2.05	0.43	0.29	0.27	0.05

^a,b,c^ Means in the same column without common letter are different at *p* < 0.05. CP = crude protein, EE = ether extract, CF = crude fiber, NFE = nitrogen-free extract, Ca = calcium, P = phosphorus, GE = gross energy, NDF = neutral detergent fiber, ADF = acid detergent fiber, ADL = acid detergent lignin. ^1^ Con = control, LAB1 = LAB 0.01% DM, LAB5 = LAB 0.05% DM, E1 = enzyme 0.01% DM, E5 = enzyme 0.05% DM, LAB1 + E1 = combination LAB 0.01% DM with enzyme 0.01% DM and LAB5 + E5 = combination LAB 0.05% DM with enzyme 0.05% DM.

**Table 4:** Nutrient composition and pH of citric acid by-product at 28 days of fermentation.

Day	Trt ^1^	CP, %	Ash, %	EE, %	CF, %	NFE, %	Ca, %	P, %	GE, MJ/kg	NDF, %	ADF, %	ADL, %	pH
28 d	Con	6.33	13.81	2.37	18.38 ^a^	51.89 ^b^	0.82	0.08	14.97	39.85 ^a^	20.08 ^a^	8.18	4.15 ^a^
	LAB1	6.20	13.93	2.44	18.03 ^a^	52.58 ^b^	0.92	0.08	14.80	40.03 ^a^	20.22 ^a^	8.74	3.92 ^b^
	LAB5	6.24	14.04	2.52	18.11 ^a^	51.63 ^b^	0.87	0.09	14.76	39.47 ^a^	19.76 ^a^	8.91	3.85 ^bc^
	E1	6.45	13.82	2.44	14.34 ^b^	55.88 ^a^	0.80	0.07	14.98	34.75 ^b^	17.66 ^a^	9.67	3.68 ^d^
	E5	6.45	13.72	2.46	14.21 ^b^	55.79 ^a^	0.81	0.07	14.93	33.96 ^bc^	17.24 ^b^	9.06	3.87 ^bc^
	LAB1 + E1	6.54	14.07	2.74	14.03 ^b^	54.76 ^a^	0.90	0.08	14.83	33.47 ^c^	17.32 ^b^	8.55	3.77 ^cd^
	LAB5 + E5	6.51	13.73	2.41	14.21 ^b^	56.49 ^a^	0.94	0.08	14.96	32.86 ^c^	16.83 ^b^	9.27	3.70 ^d^
	*p*-value	0.35	0.32	0.14	<0.001	<0.001	0.09	0.31	0.61	<0.001	<0.001	0.15	<0.001
	SEM	0.12	0.29	0.09	0.22	0.54	0.03	0.01	2.48	0.35	0.29	0.35	0.05

^a,b,c^ Means in the same column without common letter are different at *p* < 0.05. CP = crude protein, EE = ether extract, CF = crude fiber, NFE = nitrogen-free extract, Ca = calcium, P = phosphorus, GE = gross energy, NDF = neutral detergent fiber, ADF = acid detergent fiber, ADL = acid detergent lignin. ^1^ Con = control, LAB1 = LAB 0.01% DM, LAB5 = LAB 0.05% DM, E1 = enzyme 0.01% DM, E5 = enzyme 0.05% DM, LAB1 + E1 = combination LAB 0.01% DM with enzyme 0.01% DM and LAB5 + E5 = combination LAB 0.05% DM with enzyme 0.05% DM.

**Table 5:** Citric acid by-product fermented with lactic acid bacteria (LAB), enzyme, and combination of nutrient composition, gross energy (GE), and pH.

	Trt ^1^	CP, %	Ash, %	EE, %	CF, %	NFE, %	Ca, %	P, %	GE, MJ/kg	NDF, %	ADF, %	ADL, %	pH
Trt mean													
	Con	6.26 ^ab^	13.41	2.35	18.31 ^a^	52.21 ^c^	0.85	0.08	14.99	39.79 ^a^	20.36 ^a^	8.29	4.31 ^a^
	LAB1	6.22 ^b^	13.54	2.50	18.03 ^a^	51.99 ^c^	0.89	0.08	14.97	39.73 ^a^	20.04 ^ab^	8.18	4.11 ^b^
	LAB5	6.21 ^b^	13.70	2.45	18.01 ^a^	52.26 ^c^	0.86	0.08	14.99	39.82 ^a^	19.82 ^b^	8.05	4.05 ^bc^
	E1	6.33 ^ab^	13.39	2.43	16.71 ^b^	53.72 ^b^	0.85	0.08	15.00	37.79 ^b^	18.81 ^c^	8.12	4.03 ^c^
	E5	6.35 ^ab^	13.47	2.43	16.52 ^bc^	53.71 ^b^	0.86	0.08	14.94	37.19 ^c^	18.49 ^c^	8.32	4.06 ^bc^
	LAB1 + E1	6.38 ^a^	13.46	2.46	16.35 ^cd^	53.72 ^b^	0.88	0.08	14.96	37.23 ^c^	18.53 ^c^	8.15	4.03 ^c^
	LAB5 + E5	6.37 ^a^	13.36	2.43	16.13 ^d^	54.65 ^a^	0.89	0.08	14.99	36.68 ^d^	18.47 ^c^	8.11	3.97 ^d^
Day mean													
	1 d	6.25 ^b^	13.32	2.36	18.17 ^a^	52.51 ^c^	0.84	0.08	15.00	39.58 ^a^	20.01 ^a^	8.01	4.60 ^a^
	7 d	6.22 ^b^	13.39	2.45	17.83 ^b^	52.89 ^bc^	0.86	0.08	14.99	39.66 ^a^	19.88 ^a^	8.11	4.11 ^b^
	14 d	6.32 ^ab^	13.37	2.49	17.09 ^c^	53.37 ^b^	0.89	0.08	14.99	38.77 ^b^	19.32 ^b^	8.14	3.96 ^c^
	21 d	6.33 ^ab^	13.42	2.40	16.77 ^d^	52.98 ^bc^	0.88	0.08	14.95	37.24 ^c^	18.43 ^c^	8.21	3.89 ^d^
	28 d	6.39 ^a^	13.47	2.49	15.90 ^e^	54.15 ^a^	0.88	0.08	14.96	36.34 ^d^	18.44 ^c^	8.40	3.85 ^d^
**Significance of main effect and interaction**													
Trt (A)		0.04	0.17	0.49	<0.001	<0.001	0.29	0.58	0.93	<0.001	<0.001	0.62	<0.001
Day (B)		0.04	0.15	0.15	<0.001	<0.001	0.07	0.36	0.77	<0.001	<0.001	0.08	<0.001
A × B		0.97	0.66	0.83	<0.001	<0.001	0.97	0.86	0.69	<0.001	<0.001	0.77	<0.001

^a,b,c,d^ Means in the same column without common letter are different at *p* < 0.05. CP = crude protein, EE = ether extract, CF = crude fiber, NFE = nitrogen-free extract, Ca = calcium, P = phosphorus, GE = gross energy, NDF = neutral detergent fiber, ADF = acid detergent fiber, ADL = acid detergent lignin. ^1^ Con = control, LAB1 = LAB 0.01% DM, LAB5 = LAB 0.05% DM, E1 = enzyme 0.01% DM, E5 = enzyme 0.05% DM, LAB1 + E1 = combination LAB 0.01% DM with enzyme 0.01% DM and LAB5 + E5 = combination LAB 0.05% DM with enzyme 0.05% DM.

## Data Availability

Not applicable.
